# Spontaneous Hemorrhagic thyroid cyst presenting as unilateral painful thyroiditis in primary care: early recognition of a potentially life-threating complication

**DOI:** 10.1093/omcr/omag179

**Published:** 2026-10-04

**Authors:** Khaled E Elzawawi, Ibrahim Elmakaty, Gaydaa Ali, Mays Al-Krosh, Saleh M H Attal, Rasha Moussa

**Affiliations:** College of Medicine, QU Health, Qatar University, P.O. Box 2713, Doha, Qatar; Family Medicine Residency Program, Department of Medical Education, Hamad Medical Corporation, P.O. Box 3050, Doha, Qatar; Family Medicine Residency Program, Department of Medical Education, Hamad Medical Corporation, P.O. Box 3050, Doha, Qatar; Family Medicine Residency Program, Department of Medical Education, Hamad Medical Corporation, P.O. Box 3050, Doha, Qatar; Family Medicine Residency Program, Department of Medical Education, Hamad Medical Corporation, P.O. Box 3050, Doha, Qatar; Family Medicine Department, Primary Health Care Corporation, P.O. Box 26555, Doha, Qatar; Family Medicine Department, Primary Health Care Corporation, P.O. Box 26555, Doha, Qatar

**Keywords:** thyroiditis, thyroid cyst, Hemorrhagic thyroid cyst, primary care, ultrasound

## Abstract

Spontaneous hemorrhage within a thyroid nodule is an uncommon but potentially alarming condition presenting with acute neck pain, swelling, and compressive symptoms that may threaten the airway and pose diagnostic and management challenges. We describe a 35-year-old Asian woman who presented with sudden onset of severe left-sided neck pain and swelling, accompanied by dysphagia and exertional dyspnea. There was no history of trauma, anticoagulant use, or prior thyroid intervention. Physical examination revealed marked unilateral thyroid tenderness and swelling without overt airway compromise. Thyroid function tests were within normal limits. Neck ultrasonography demonstrated a complex mixed solid–cystic left thyroid nodule measuring 3.2 × 2.4 × 3.5 cm with internal echogenic septations, consistent with a hemorrhagic thyroid cyst. Initial conservative management with non-steroidal anti-inflammatory drugs provided minimal relief. The patient was subsequently treated with a short course of high-dose oral corticosteroids and underwent ultrasound-guided fine-needle aspiration, yielding 7 mL of dark brown hemorrhagic fluid. Cytological examination revealed macrophage-predominant cyst contents with no evidence of malignancy. Significant symptomatic improvement was noted following aspiration and steroid therapy. The patient was referred for endocrinology follow-up, with elective hemithyroidectomy discussed due to the potential risk of recurrence. Spontaneous hemorrhage of a thyroid nodule, although rare, should be considered in patients presenting with acute unilateral neck pain and swelling, even in the absence of identifiable risk factors. Early imaging, vigilant airway assessment, and individualized management are crucial. In clinically stable patients, conservative treatment with aspiration and corticosteroids may be effective, while surgical intervention should be reserved for recurrent cases or those with airway compromise. This case highlights the critical role of primary care physicians in early recognition and urgent referral of rare but potentially life-threatening thyroid conditions presenting with acute neck swelling.

## Teaser

1. Spontaneous intranodular hemorrhage can present with acute neck pain and compressive symptoms threatening the airway even without identifiable risk factors.
2. Hemorrhagic thyroid nodules may mimic other neck emergencies, requiring urgent treatment, careful evaluation and airway assessment.
3. Ultrasound plays a key role in early diagnosis by identifying complex cystic features suggestive of hemorrhage.
4. Ultrasound-guided aspiration combined with short-course corticosteroids can provide significant symptomatic relief in stable patients.
5. Emergent early referral to emergency and endocrinology is essential for timely evaluation and intervention, particularly in patients with potential airway compromise.

## Introduction

Thyroid cysts and nodules are common in the general population and are frequently detected incidentally during clinical or radiological evaluation [1, 2]. While most thyroid cysts are benign and asymptomatic, a rarer complication known as intranodular hemorrhage infrequently occurs when acute bleeding into a pre-existing thyroid spontaneously starts without precipitating factors such as trauma or the use of anticoagulants [3–5]. This becomes known as spontaneous hemorrhagic thyroid cyst and may lead to acute neck swelling and compressive symptoms, posing diagnostic and management challenges [3–5]. The development of spontaneous hemorrhagic cyst of the thyroid is an uncommon clinical entity, typically associated with precipitating factors such as trauma, anticoagulation, or recent fine-needle aspiration [3, 4]. Truly spontaneous cases, occurring in the absence of identifiable risk factors, are particularly rare and may mimic infectious, neoplastic, or vascular conditions of the neck. Prompt recognition is essential, as rapid expansion can result in airway compromise [6, 7].

We report a case of spontaneous thyroid hemorrhagic cyst presenting with acute neck swelling and progressive dysphagia in the absence of recognized precipitating factors. This case highlights the importance of early imaging, vigilant airway assessment, and individualized management strategies in achieving favorable outcomes.

## Case report

A 35-year-old Vietnamese woman presented to our walk-in primary care clinic with a four-day history of spontaneous, progressive left-sided anterior neck swelling associated with severe pain. The pain was rated as 9/10 in severity, non-radiating, and not relieved or exacerbated by movement. She denied any preceding trauma, recent invasive neck procedures, or anticoagulant use. The swelling was accompanied by dysphagia to both solids and liquids, as well as exertional dyspnea during mild activities such as hair combing and when lying supine. There was no history of fever, upper respiratory tract infection, or voice change.

The patient reported associated nonspecific symptoms including fatigue, cold intolerance, dry skin, and increased hair loss, along with an unintentional weight gain of approximately 5 kg over recent months. She also described increased sleep duration and persistent daytime somnolence. Gynecological history revealed menstrual irregularity over the preceding year, with menses occurring every two to three months. She denied changes in bowel habits. This was her first episode of neck swelling. She had no past medical or surgical history and no family history of thyroid disease or thyroid malignancy. She did not take regular medications or supplements. The patient reported a remote history of a generalized pruritic rash following amoxicillin administration four years earlier, without associated respiratory symptoms.

On examination, the patient was hemodynamically stable, with a blood pressure of 98/65 mmHg, heart rate of 70 beats per minute, respiratory rate of 17 breaths per minute, and a body mass index of 23.5 kg/m^2^. Inspection revealed an obvious left-sided anterior neck swelling as seen in Figs 1 and 2. Palpation demonstrated a soft, markedly tender unilateral neck mass that did not move with swallowing, without overlying skin changes or cervical lymphadenopathy. No bruit was appreciated on auscultation. Respiratory examination was unremarkable, and there were no signs of acute airway compromise.

**Figures 1 and 2:**
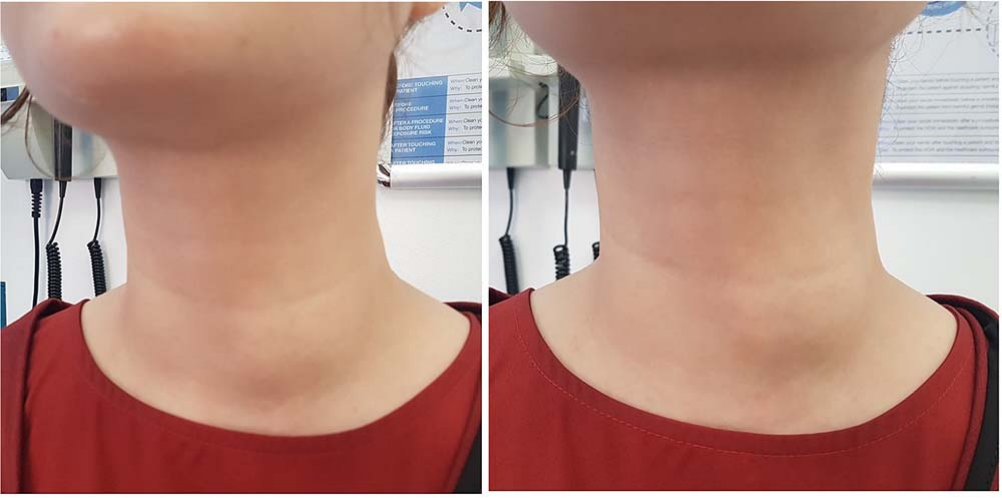
Anterior view of the patient’s neck demonstrating visible swelling consistent with thyroid enlargement on initial examination.

Neurological examination revealed a fine hand tremor when the patient extended her arms, noted using the paper test. Deep tendon reflexes were brisk in the upper limbs, more pronounced on the left, and asymmetric hyperreflexia was noted in the right lower limb at the patellar reflex. Cranial nerve examination was otherwise unremarkable, with no lid lag and a normal H-test. The remainder of the physical examination was unremarkable.

Laboratory investigations demonstrated normal thyroid function, with a thyroid-stimulating hormone level of 2.73 mIU/L and free thyroxine of 16.4 pmol/L (Table 1). Full blood count, renal and liver function tests, inflammatory markers, and peripheral smear were within normal limits. Lipid profile showed elevated total cholesterol and low-density lipoprotein levels. Vitamin D deficiency was noted. A urine pregnancy test was negative. No coagulation abnormalities were identified.

**Table 1:** Blood investigation at initial evaluation.

Category	Test	Result
**Basic Metabolic Panel**	Creatinine	68 μmol/L
	eGFR	>90 mL/min/1.73m^2^
	Urea	3.2 mmol/L
**Liver Function Tests**	Albumin	42 g/L
	Alk Phos	62 U/L
	ALT	14.7 U/L
	AST	20 U/L
	Total Bilirubin	8.5 μmol/L
	Total Protein	74 g/L
**Hematology**	Hemoglobin (Hgb)	12.3 g/dL
	Hematocrit (Hct)	38.2%
	RBC	4.1 × 10^6^/μL
	WBC	9.1 × 10^3^/μL
	Platelets	237 × 10^3^/μL
	MPV	10.1 fL
	RDW-CV	12.1%
	ESR	19 mm/hr
	INR	—
**Differential Count**	Neutrophils	58.5% (5.3 × 10^3^/μL)
	Lymphocytes	32.6% (3.0 × 10^3^/μL)
	Monocytes	6.9% (0.6 × 10^3^/μL)
	Eosinophils	1.3% (0.12 × 10^3^/μL)
	Basophils	0.7% (0.06 × 10^3^/μL)
**RBC Indices**	MCV	94.1 fL
	MCH	30.3 pg
	MCHC	32.2 g/dL
**Lipid Panel**	Total Cholesterol	6.4 mmol/L
	LDL (calculated)	4.8 mmol/L
	HDL	1.3 mmol/L
	Non-HDL	5.1 mmol/L
	Triglycerides	0.7 mmol/L
**Endocrine/Thyroid**	TSH	2.73 mIU/L
	Free T4 (FT4)	16.4 pmol/L
	Vitamin D	18 ng/mL
**Other**	Peripheral Smear	Normal
	Urine Pregnancy Test	Negative

Initially, as the patient was hemodynamically stable and exhibited no life-threatening symptoms, conservative management was initiated. She was treated symptomatically with intravenous diclofenac followed by a one-week course of high-dose oral nonsteroidal anti-inflammatory drugs, and an urgent thyroid ultrasound was arranged. Despite this approach, there was no clinical improvement after one week and symptoms actually worsened.

Ultrasonography of the thyroid gland demonstrated a normal right thyroid lobe with homogeneous echotexture, measuring 1.5 × 1.4 × 4.5 cm (volume approximately 5.3 cc), with no focal solid or cystic lesions and no pathological calcifications. The left thyroid lobe was enlarged, measuring 2.2 × 3.1 × 5.9 cm (volume approximately 22.0 cc), with preserved overall echotexture. Two nodules were identified within the left lobe. The upper pole contained a small mixed solid–cystic nodule measuring 0.9 × 0.8 × 1.0 cm (0.5 cc). The lower pole demonstrated a larger cystic nodule measuring 3.2 × 2.4 × 3.5 cm (14.5 cc), characterized by a fluid–fluid level and internal echogenic septations, findings consistent with a hemorrhagic thyroid cyst (Figures 3 and 4). No suspicious calcifications were identified. The thyroid isthmus measured 0.2 cm in anteroposterior diameter and was unremarkable. There was no evidence of retrosternal extension, abnormal cervical lymphadenopathy, or vascular abnormality. Overall findings were classified as TI-RADS 2. Thyroiditis was considered as the primary initial differential diagnosis but was then ruled out due to the relatively low erythrocyte sedimentation rate (19 mm/hr), absence of systemic inflammatory symptoms, unusual persistence and worsening of compressive symptoms, increase neck size, and U.S imaging findings consistent with a hemorrhagic cyst. Thyroid antibody testing was not performed, which represents a limitation in fully excluding autoimmune thyroid conditions.

**Figures 3 and 4:**
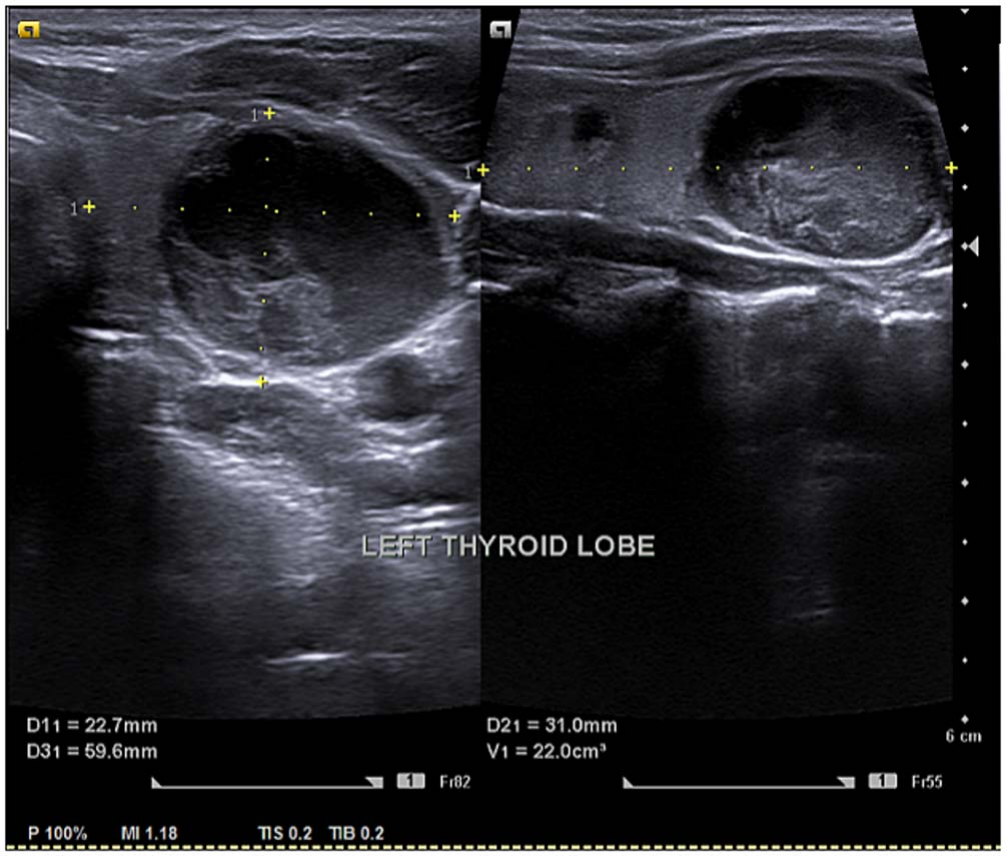
Ultrasound images of the left thyroid lobe demonstrating a normal-sized left thyroid lobe with homogeneous echotexture. A cystic nodule was identified in the lower pole of the left lobe, measuring 3.2 × 2.4 × 3.5 cm (estimated volume 14.5 mL). The nodule demonstrated a fluid–fluid level and internal echogenic septations, consistent with a hemorrhagic thyroid cyst. No suspicious calcifications were detected.

Given the persistence of symptoms, a therapeutic course of oral prednisolone (50 mg daily) was initiated, and the patient was referred to endocrinology for ultrasound-guided fine-needle aspiration (FNA) of the lesion. Aspiration yielded approximately 7 mL of dark brown fluid composed predominantly of macrophages, consistent with hemorrhagic cyst contents and demonstrating a low risk of malignancy.

At one-week follow-up after aspiration and completion of the steroid course, the patient reported partial symptomatic improvement. In view of the risk of recurrence, she was subsequently referred to endocrinology for further evaluation and consideration of left hemithyroidectomy.

The rationale for giving the patient corticosteroids was to potentially decrease any inflammation in the area that may have been causing the swelling. Moreover, although the lesion was classified as TI-RADS 2, suggesting a benign nature, FNA was done due to the acute onset, rapid progression, and associated compressive symptoms, raising concern for hemorrhage and potential airway compromise and possible malignancy at the time.

## Discussion

Thyroid cysts are common clinical findings, with a reported prevalence ranging from 19% to 68% in the general population and increasing markedly with age, reaching nearly 80% in individuals older than 80 years [8]. The vast majority of thyroid cysts are benign and asymptomatic and are therefore managed conservatively. In contrast, spontaneous hemorrhage within a thyroid cyst is an uncommon complication that may present acutely and pose significant diagnostic and therapeutic challenges, particularly because of the potential for airway compromise [6].

Hemorrhagic thyroid cysts are most frequently associated with identifiable precipitating factors, including blunt cervical trauma, recent FNA, anticoagulation therapy, or mechanical stress [6, 7, 9]. Truly spontaneous intracystic hemorrhage, occurring in the absence of these recognized risk factors, is exceedingly rare, and its underlying etiology remains incompletely understood [9]. Patients typically present with sudden-onset anterior neck pain and swelling, often accompanied by dysphagia, voice changes, or respiratory distress due to rapid intrathyroidal expansion and compression of adjacent structures [6, 7]. Despite the dramatic presentation, only a small proportion of patients—estimated at 0.7% to 1.59%—ultimately require surgical intervention, with management decisions guided primarily by symptom severity and airway involvement [9].

Several pathophysiological mechanisms have been proposed to explain spontaneous hemorrhage within thyroid cysts. Anatomical and pathological studies suggest that venous bleeding into the cyst wall or adjacent adenomatous tissue is the most likely source [10]. Capsular and intracystic veins often lack a well-developed elastic layer, rendering them vulnerable to rupture at relatively low intravascular pressures, whereas intrathyroidal arteries are structurally more resilient [10]. Additional contributing factors may include dilated lobular vessels, increased intracystic vascularity, and the presence of arteriovenous shunts, which can expose fragile venous structures to elevated pressures [11]. Transient increases in venous pressure, such as those occurring during coughing, straining, physical exertion, or Valsalva maneuvers, have been repeatedly implicated as triggering events in otherwise spontaneous cases [11].

Published case reports further support these mechanisms. Cerna et al. described a large arteriovenous malformation of the thyroid gland resulting in airway compression that required embolization followed by surgical resection, while Szeto et al. reported acute rupture of a thyroid cyst immediately following endotracheal intubation [7]. In contrast, many reported cases—including the present one—demonstrate no identifiable precipitating factors such as trauma, anticoagulation, or prior instrumentation, supporting the diagnosis of true spontaneous intracystic hemorrhage [6, 9].

From a diagnostic perspective, spontaneous thyroid hemorrhage represents a rare but potentially life-threatening cause of acute neck swelling and may clinically overlap with other cervical emergencies [12, 13]. Early evaluation should prioritize airway stability, as rapidly expanding thyroid hematomas have been reported to cause acute airway compromise requiring urgent intervention [12, 13]. Careful clinical assessment is essential to distinguish hemorrhagic thyroid cysts from infectious processes such as deep neck space abscesses or suppurative thyroiditis, which typically present with fever, skin erythema, and elevated inflammatory markers [12].

Laboratory investigations are generally nonspecific [9]. Thyroid function tests are usually normal, although transient thyrotoxicosis has been reported secondary to hormone release following intracystic bleeding [9]. In part, this thyrotoxicosis may have been responsible for some of our patient’s symptoms in this case including fine hand tremors, brisk upper limb reflexes, and asymmetric lower limb hyperreflexia. Moreover, Valenta, L. J., & Elias, A. N. (1983) explain how some patients can clinical present hypothyroidism symptoms, sometimes severe in nature, while retaining normal thyroid function tests in labs [14]. In addition, they show and explain that, in such unclear cases, the use of a more sensitive diagnostic thyrotropin-releasing hormone (TRH) test is of greater accuracy in pinpointing thyroid dysfunction [14]. Additionally, this further proves that, just like in our case report, normal basic thyroid function tests (TSH, T4) do not necessarily fully rule out thyroid disease which is a major takeaway lesson. Perhaps, this could guide primary care physicians encountering a similar case in the future by preventing them from ruling out thyroid disease only because of normal thyroid function tests. In addition, it would make them better prepared and equipped for the next step which is TRH testing in such cases. Finally, whether this unspecific paradoxical clinical presentation is due to the nature of spontaneous hemorrhagic thyroid cysts itself or was due to other pathophysiological processes all happening at the same time is something that needs to be further examined in future research.

Coagulation studies may help identify underlying bleeding disorders; however, many spontaneous cases occur in patients without demonstrable coagulopathy [5]. Fine-needle aspiration is often deferred during the acute phase because of the potential risk of exacerbating bleeding and its limited diagnostic yield in the presence of a hematoma [9]. Ultrasonography remains the recommended first-line imaging modality as it is relatively fast and non-invasive. Hemorrhagic thyroid cysts typically appear as heterogeneous or complex cystic lesions containing internal echoes consistent with blood products. Doppler imaging is useful for excluding vascular lesions or hypervascular tumors and for differentiating hemorrhage from inflammatory or malignant pathology. Computed tomography (CT) serves as a valuable adjunct when ultrasound findings are inconclusive or when there is concern for airway compression, retrosternal extension, or involvement of adjacent structures. CT allows accurate delineation of hematoma size and extent and facilitates differentiation of thyroid hemorrhage from traumatic, vascular, or malignant causes of acute neck swelling.

Management strategies depend on clinical stability and symptom severity. In most reported cases, spontaneous hemorrhage within a thyroid cyst does not necessitate emergent thyroidectomy. A large retrospective emergency department study involving over 630 000 adult patients identified only 59 cases of acute hemorrhage into a thyroid cyst, highlighting the rarity of this entity [5]. Most patients were managed conservatively, with only one requiring urgent surgery for uncontrolled bleeding and approximately 10% requiring hospital admission. Delayed FNA performed after symptom resolution demonstrated predominantly benign or nondiagnostic cytology, and no significant complications such as recurrent hemorrhage or infection were reported, supporting a generally favorable prognosis with appropriate follow-up [5].

When patients are hemodynamically stable and without airway compromise, elective surgical management—typically thyroid lobectomy or total thyroidectomy with intraoperative neuromonitoring—remains the definitive treatment option, particularly in cases with persistent symptoms or concern for recurrence [9]. Conservative approaches have also been described. Chan *et al*. reported successful nonoperative management of a 93-year-old patient using needle aspiration and intravenous corticosteroids, resulting in symptom resolution without surgery [15]. Conversely, emergent surgical intervention may be unavoidable in unstable patients. Lee et al. described a case of acute airway obstruction secondary to spontaneous hemorrhage into a thyroid cyst requiring emergency subtotal thyroidectomy, underscoring the potentially life-threatening nature of this condition [16].

Although spontaneous hemorrhage into thyroid cysts usually follows a benign course, rare fatal outcomes have been reported. Dell’Aquila *et al*. described a fatal case in which massive hemorrhagic enlargement of a multinodular goiter resulted in external tracheal compression and death [17]. This highlights the importance of early recognition, vigilant airway assessment, and individualized management to prevent catastrophic outcomes.

As this is a single case report, it is only fair to assume that its findings are subject to inherent limitations, including limited generalizability to the public. Additionally, the favorable outcome observed in this case may not be representative of all patients with similar conditions as is also seen in other case reports [17].

All of these factors make this case not only worth reporting but also of extreme significance especially from a family medicine and primary care point of view. While the management of spontaneous hemorrhagic thyroid cyst has been described in more specialized settings, this case underscores the importance of early recognition in primary care, where patients may first present with non-specific or rapidly evolving symptoms. In such settings, physicians must rely on clinical judgment to identify red flags, initiate appropriate initial evaluation, and arrange urgent referral to prevent airway compromise and other serious life-threatening complications. To further elaborate, thyroid disorders, including thyroid cysts and nodules, are some of the most common conditions seen at the level of primary care and managed by family medicine physicians [8, 18–20]. Thus, the significance of this case lies in how it highlights the weight of timely management and emergent referral in the primary care when spontaneous hemorrhagic thyroid cyst is suspected, given the potential for life-threatening airway compromise if not promptly managed and the high chance of similar patient presentation to primary care.

Diagnosis can be challenging due to the non-specific presentation, which may mimic other conditions such as thyroiditis or subacute thyroiditis especially post viral infection, benign thyroid cysts or nodules, neck abscesses, or malignancy. In primary care settings, this overlapping presentation of symptoms may lead to delayed recognition, particularly in the absence of classic compressive symptoms.

The case was selected due to its high clinical relevance, particularly the acute presentation and potential for life-threatening complications requiring urgent intervention. We acknowledge the inherent selection bias in case reports, as unusual or severe presentations are more likely to be reported. However, this case was chosen to highlight important diagnostic and management considerations relevant to primary care practice. This case may impact clinical practice by reinforcing the need for heightened awareness of rare but potentially life-threatening thyroid conditions in primary care, encouraging early recognition and timely referral. In terms of future research, further studies are needed to better characterize the presentation, optimal management strategies, and outcomes of spontaneous hemorrhagic thyroid cysts, particularly in outpatient and primary care settings. Another significant area that needs to be studies as well is patients’ precipitating risk factors for that lead to spontaneous hemorrhagic thyroid cysts. All clinical data were collected from patient interviews, physical examination findings, laboratory investigations, imaging studies, and follow-up records.

## Data Availability

All data generated or analyzed during this study are included in this published article.
